# Conventional Study on Novel Dicationic Ionic Liquid Inclusion with β-Cyclodextrin

**DOI:** 10.3390/ijms12096329

**Published:** 2011-09-23

**Authors:** Sharifah Mohamad, Hemavathy Surikumaran, Muggundha Raoov, Tilagam Marimuthu, Kumuthini Chandrasekaram, Puvaneswary Subramaniam

**Affiliations:** 1 University of Malaya Centre for Ionic Liquids, Department of Chemistry, Faculty of Science, University of Malaya, Kuala Lumpur 50603, Malaysia; E-Mails: hems8620@yahoo.com (H.S.); vainavi38@gmail.com (T.M.); kumuthinic@um.edu.my (K.C.); puva@um.edu.my (P.S.); 2 Advanced Medical & Dental Institute, University of Science Malaysia, No 1–8 (Lot 8), Persiaran Seksyen 4/1, Bandar Putra Bertam, Kepala Batas, Pulau Pinang 13200, Malaysia; E-Mail: muggundha_raoov@hotmail.com

**Keywords:** dicationic ionic liquid, cyclodextrin, inclusion complex

## Abstract

This study focuses on the synthesis and characterization of the inclusion complex of β-Cyclodextrin (β-CD) with dicationic ionic liquid, 3,3′-(1,4-Phenylenebis [methylene]) bis(1-methyl-1*H*-imidazol-3-ium) di(bromide) (PhenmimBr). The inclusion complex was prepared at room temperature utilizing conventional kneading technique. Proton (^1^H) NMR and 2D (^1^H–^1^H) COSY NMR were the primary characterization tools employed to verify the formation of the inclusion complex. COSY spectra showed strong correlations between protons of imidazolium and protons of β-CD which indicates that the imidazolium ring of PhenmimBr has entered the cavity of β-CD. UV absorption indicated that β-CD reacts with PhenmimBr to form a 2:1 β-CD-PhenmimBr complex with an apparent formation constant of 2.61 × 10^5^ mol&^−2^ L^2^. Other characterization studies such as UV, FT-IR, XRD, TGA, DSC and SEM studies were also used to further support the formation of the β-CD-PhenmimBr inclusion complex.

## 1. Introduction

Supramolecular chemistry is a discipline of chemistry which has been attracting much attention recently, especially the host-guest type interaction. Among all the potential hosts, Cyclodextrin (CD) is the most significant one [1]. CDs are well known series of macro cyclic oligosaccharides resulting from the degradation of starch by bacterial enzymes [2]. Generally, CDs are composed of 6, 7, or 8 glucose units connected by α-1,4-glucosidic linkages which are categorized as α-, β- and γ-CD, respectively [3–6]. Among these CDs, β-Cyclodextrin (β-CD) has been chosen for this study since its cavity size is suitable for guest molecules with molecular weights between 200 and 800 g/mol [7,8]. β-CD can accommodate variety of organic and inorganic compounds to form inclusion complexes (ICs) due to its hydrophobic cavity [9,10] thus it is being widely used in pharmaceutical industry [11], foodstuff [12,13], separation studies [14] and environmental engineering [15,16].

An ionic liquids (IL), is a salt in which the ions are poorly coordinated. Consequently these compounds are liquid below 100 °C or even at room temperature (RTIL’s). They remain in the liquid state over a wide temperature range, and have unique properties such as non-volatility, non-flammability, low viscosity, chemical and electrochemical stability [17]. Recently, ionic liquid is being considered as a new and remarkable class of solvents as they are good solvents for a wide range of organic and inorganic materials [18]. According to Wilkes the recent explosion of interest in the ILs [19] is due to the realization that these materials, used mostly for specialized electrochemical applications may have greater utility as reaction solvents. In comparison with monocationic ILs, multifunctional ILs (dicationic and dianionic ILs) have potential to exhibit a greater range of physical properties [20] in terms of greater thermal stability, lower volatility, and more flexibility in tuning/varying their physicochemical properties.

Understanding the interaction between ILs and CDs is important to analytical chemistry and material synthesis [21–24]. It was found that ILs could solubilize a number of complex organic molecules such as CDs which is very useful in separation community. Due to high solubility with CDs, ILs has been providing additional selectivity and resolution for separations. Successful separation with the combination of CDs and ILs suggests that there may be interactions between CDs and ILs. Therefore study of inclusion complex between CDs and ILs is very important to investigate the actual nature of the interaction. There are several reports on CDs with monocationic ILs [8,10,25,26–29]. To the best of our knowledge, studies on the inclusion complexation of dicationic IL with β-CD in the scientific literature are scarce [30]. Hence in this study, the binding nature of the dicationic IL (PhenmimBr) with β-CD is analysed and compared with monocationic ILs.

## 2. Experimental

### 2.1. Reagent and Solution

β-CD is commercially available and was purchased from Acros (Hungary) (99%). α, α-dibromo-*p*-xylene and 1-methylimidazole was supplied by Merck. Other reagents and chemicals were of analytical reagent grade and were used as received without further purification. Double distilled water was used throughout the experiment.

### 2.2. Instrumentation

The Proton Nuclear Magnetic Resonance ^1^H (NMR) and Correlation Spectroscopy (COSY) spectra of the samples in dimethyl sulfoxide (DMSO) were recorded on Lambda JEOL 400 MHz Fourier Transform NMR (FT-NMR) spectrometer at room temperature. Calibration of proton chemical shift was achieved by using tetramethylsilane (TMS) as an internal reference standard. The Fourier Transform Infrared (FT-IR) spectra were recorded on a Perkin–Elmer RX1 FT-IR spectrometer with samples prepared using potassium bromide (KBr) pellets. All the samples were run in the spectral region range of 400–4000 cm^−1^. X-ray diffraction (XRD) patterns were taken using Cu Kα irradiation with a Siemens D5000 X-ray diffractometer (voltage, 40 kV; current, 100 mA). Powder samples were mounted on a sample holder and scanned from 5° to 30° at a speed of 3° per minute. Thermo gravimetric analyses (TGA) curves were examined using a TA Instruments Q500. A linear heating rate was set at 20 °C per minute within the temperature range from 50 °C to 900 °C in a stream of nitrogen atmosphere. Differential Scanning Calorimetry (DSC) analysis was done by heating the samples from 36 °C to 350 °C at 5 °C per minute. Spectrophotometric measurements were made with a Shimadzu Ultraviolet-Visible spectroscopy (UV–Vis) recording spectrophotometer equipped with 1 cm quartz cells.

### 2.3. Synthesis

#### 2.3.1. Preparation of 3,3′-(1,4-Phenylenebis [methylene]) bis(1-methyl-1*H*-imidazol-3-ium) di(bromide) (PhenmimBr)

**Figure 1:**
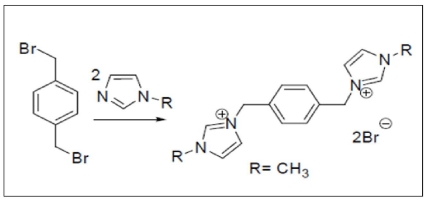
Synthesis of PhenmimBr.

PhenmimBr was prepared as reported previously by Kilivelu and Yatimah [31]. The mixture of α, α-dibromo-*p*-xylene and 1-methylimidazole (1:2) were dissolved in acetonitrile and the solution refluxed at 60 °C. The reaction product was allowed to cool to room temperature and deposit overnight. The resulting white precipitate was collected by vacuum filtration, washed with acetone several times and dried in a vacuum oven. The end product obtained was a white powder with the calculated yield of 72%. The reaction mechanism for the synthesis of PhenmimBr from the starting materials, 1-methylimidazole and α, α-dibromo-*p*-xylene is shown in Figure 1. The reaction mechanism shows that the PhenmimBr is produced by a simple aromatic nucleophilic substitution reaction.

#### 2.3.2. Synthesis of β-Cyclodextrin-PhenmimBr (β-CD-PhenmimBr)

The inclusion complex of β-CD with PhenmimBr was prepared using conventional kneading method [32]. Equimolar amounts of β-CD and PhenmimBr was kneaded with mortar and pestle in minimal ethanol to form a homogeneous paste. The complex was kneaded for approximately 30 minutes and dried to constant mass. After drying, a white powder (β-CD-PhenmimBr complex) was obtained. The calculated yield was 78%.

### 2.4. Procedure

#### 2.4.1. Preparation of β-CD-PhenmimBr for Spectroscopic Studies

A 2.0 mL portion of 0.01 mM PhenmimBr aliquot and 3.2 mL of 0.0032 M β-CD solution was adjusted to pH 7.0, transferred accurately into a 10.0 mL standard volumetric flask and diluted to the mark with double distilled water. The absorption spectrum of β-CD-PhenmimBr complex was recorded against blank reagent which was prepared with the same reagent concentration but without the addition of PhenmimBr. In addition, absorption spectra of PhenmimBr and β-CD were also recorded. All the absorbance was measured at 200 nm separately against blank reagent. For the formation constant curve, the concentration of PhenmimBr was held constant at 0.01 mM, meanwhile the concentration of β-CD was varied (0, 0.001, 0.002, 0.003 and 0.004 M). This procedure was replicated in order to obtain three or more absorbance values for each of the β-CD concentration studied.

## 3. Results and Discussion

### 3.1. ^1^H Nuclear Magnetic Resonance (NMR) Spectra

**Figure 2:**
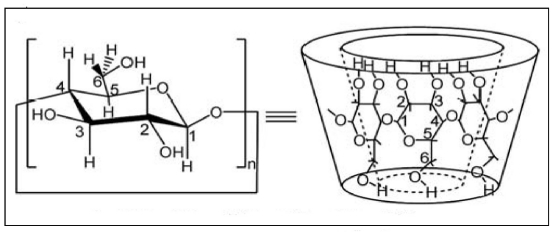
Assignation of protons in β-CD.

**Figure 3:**
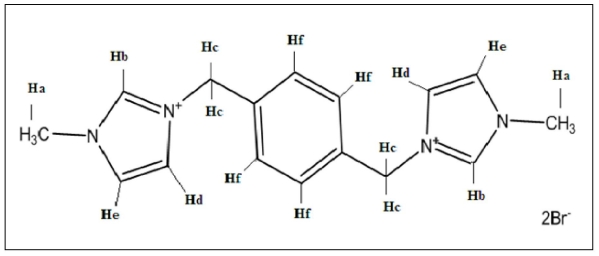
Structure of PhenmimBr with labeled protons.

^1^H NMR spectra is a useful technique to confirm the formation of an inclusion complex and can provide useful information on the inclusion mechanism of CDs with the guest molecules. Chemical shift changes of specific nuclei in the host molecule can verify the formation of inclusion complex in solution, since significant changes in microenvironment are known to occur in CD of the inclusion complex [32]. The obvious upfield shift of the protons on the inner cavity of β-CD, *i.e*., H3 and H5 were observed due to anisotropic shielding by ring current from the aromatic rings of PhenmimBr (Table 1). The H1, H2 and H4 protons of β-CD, on the outer part of the cavity (Figure 2) also faces an upfield shift but do not exhibit considerable changes upon addition of PhenmimBr. The same phenomenon is observed with the H6 proton. From the ^1^H NMR data, it can also be surmised that when PhenmimBr enters the hydrophobic cavity of β-CD, the change of the micro-environment in PhenmimBr protons lead to the upfield shift of the protons. Hb and Hd protons of the imidazolium ring (Figure 3) shows obvious shift upon inclusion complex formation. The presence of ^1^H signals belonging to both β-CD and PhenmimBr molecules could be observed in ^1^H NMR spectrum of β-CD-PhenmimBr which strongly suggests that the new inclusion complex has been formed.

### 3.2. Correlation Spectroscopy (COSY)

**Figure 4:**
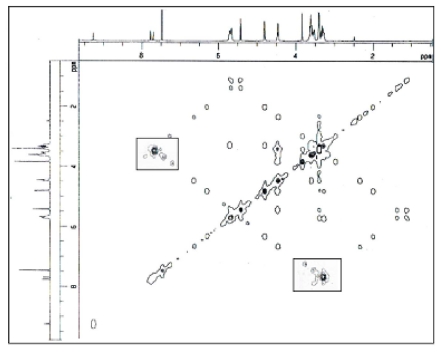
Correlation Spectra of β-CD-PhenmimBr in DMSO.

To obtain further information of this complex, 2D ^1^H–^1^H COSY spectroscopic technique was studied. 2D NMR is a powerful tool for investigating intermolecular interaction and to gain more information on the conformation of the inclusion complex [33]. The 2D COSY spectrum is essential in determining the interaction between the guest and the host molecules in the complexes and the connectivity between neighboring protons in the inclusion complex [34]. The cross-peaks in the spectra, indicated in Figure 4 originate from the interaction of the protons of PhenmimBr and β-CD. The cross peaks of β-CD (3.5–3.6 ppm, H-3, H-5) and PhenmimBr (7.6–7.8 ppm, H-d, e) demonstrates strong intensity. The strong correlation observed suggests that the imidazolium rings of PhenmimBr are strongly interacting with β-CD. Hence, from the COSY spectra we can conclude that the imidazolium rings of PhenmimBr have entered the cavity of the β-CD.

### 3.3. Fourier Transform Infrared (FT-IR) Spectroscopy

**Figure 5:**
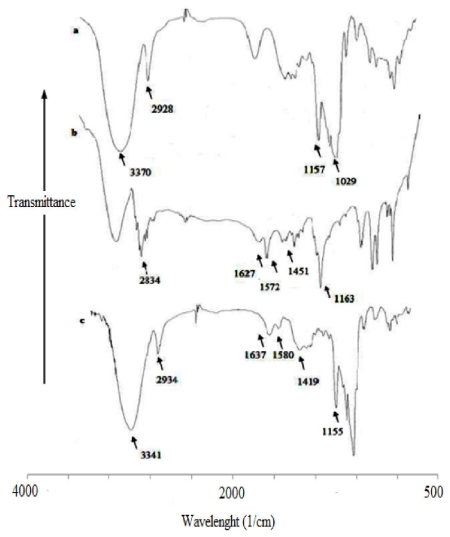
Fourier transform infrared spectra of (**a**) β-CD; (**b**) PhenmimBr; and (**c**) β-CD-PhenmimBr.

FTIR is another useful technique to confirm the formation of an inclusion complex. The FTIR spectrum of β-CD-PhenmimBr is similar to that of pure β-CD, which is a major characteristic for the host-guest inclusion complex of CDs [10]. The most obvious bands: CH_3_ (N) stretch, C=N, imidazolium ring bend (C=C) and CH_2_ bend of PhenmimBr were observed in the FTIR spectrum of the β-CD-PhenmimBr at 2934, 1637, 1580 and 1419 cm^−1^ respectively (refer to Figure 5c). Generally, the intensity and shape of the bands in β-CD-PhenmimBr were shifted upon inclusion complexation compared to the bands in the free PhenmimBr. The broad −OH stretching band of the β-CD at 3370 cm^−1^ corresponds to the multiple −OH functional groups in β-CD molecules [35] as shown in Figure 5(a). This broad hydroxyl (−OH) band narrows in the spectrum of β-CD-PhenmimBr (3341 cm^−1^). Apart from that, CH_2_ bend of PhenmimBr (1451cm^−1^) is reduced and shifts upon formation of β-CD-PhenmimBr (1419 cm^−1^). In addition 1163 cm^−1^ absorption peak of C–N was also reduced and shifts in β-CD-PhenmimBr (1155 cm^−1^). From FT-IR spectra a conclusion can be made, that the formation of inclusion complex between β-CD and PhenmimBr occurs with non-covalent interactions such as hydrophobic interactions, van der Waals interactions and hydrogen bonds which lowers the energy of the included part of PhenmimBr and hence reduces the absorption intensities of the corresponding bonds. The introduction of CD molecules into the IL system occurs with a disruption of the hydrogen bond network which makes ILs potential key tool in the preparation of a new generation of chemical nanostructures [36].

### 3.4. X-ray Diffraction (XRD) Analysis

**Figure 6:**
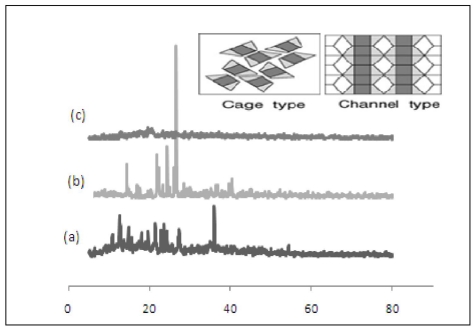
X-ray diffractogram of (**a**) β-CD; (**b**) PhenmimBr; and (**c**) β-CD-PhenmimBr.

X-ray diffraction (XRD) provides further evidence for the formation of inclusion structure and it is widely used to determine the formation of a new compound from their parent molecules [35]. Figure 6 shows the XRD patterns of pure β-CD, PhenmimBr and β-CD-PhenmimBr. Generally, the crystal structures of CD complexes are categorized mainly into three types: cage-type, channel-type and layer-type. The β-CD-PhenmimBr obtained was a fine crystalline powder. The results suggested that the XRD pattern of the β-CD-PhenmimBr was a head-to-head channel type structure [37]. Referring to Figure 6c, the XRD pattern of the β-CD-PhenmimBr is totally different from the XRD patterns of its parent molecules and this confirms the formation of the inclusion complex. In Figure 6a, major peaks appear at 9.5°, 11.1°, 12.8°, 13.3°, 18.8° and 21.8° indicating that β-CD adopted a typical cage structure. While PhenmimBr Figure 6b, shows a typical channel type structure with major peaks appearing at 14.2°, 22.3°, 24.2° and 27.5°. Generally, CD molecules are stacked along an axis to form a cylinder for the inclusion complex adopting a head-to-head channel-type structure [10]. The difference between both diffractrograms is due to the interaction between β-CD and PhenmimBr, confirming the formation of the inclusion complex.

### 3.5. Thermogravimetric Analysis (TGA)

**Figure 7:**
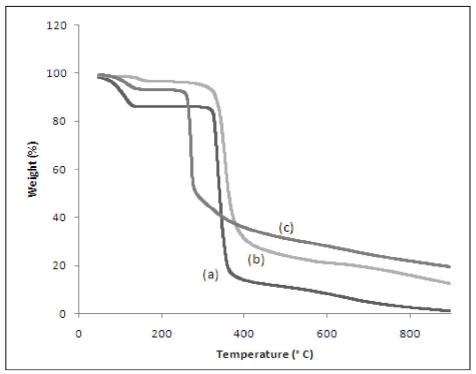
Thermogravimetric analysis curves of (**a**) β-CD; (**b**) PhenmimBr; and (**c**) β-CD-PhenmimBr.

TGA is a frequently used technique to measure a compound’s thermal stability. In this study, the thermal stability of β-CD-PhenmimBr was evaluated using TGA and the results were compared with pure β-CD and free PhenmimBr. Figure 7 shows the weight-loss curves for β-CD-PhenmimBr and its precursors. β-CD starts to decompose at 320 °C. Visible changes occurred at 120 °C due to the endothermic behavior which corresponds to loss of water molecules in β-CD cavity. PhenmimBr starts to decompose at 330 °C. But β-CD-PhenmimBr starts to decompose at 280 °C, which means the inclusion complex has lowered the initial decomposition temperature of both β-CD and PhenmimBr. According to Okumura *et al.* usually the initial decomposition temperature of CD complexes is higher than those of CD and the inclusion complexation is supposed to contribute to better stability of CDs [37]. However, the current TGA results are very unusual. This phenomenon may result from the unique molecular structure and properties of the dicationic IL compared to organic compounds and polymers. The steric congestion and the geometry distortion of the components may affect the stability and result in reduction of thermal stability of β-CD-PhenmimBr [10]. Moreover, when the cation of IL is incorporated within the cavity of β-CD, the distance between the cation and anion become larger than in the ion pair, which may lower the decomposition temperature of the complex [25]. Similar phenomenon has been observed in our previous work in which dicationic IL, 1,1′,2,2′-tetramethyl-3,3′-*p-*phenylenedimethylene diimidazolium dibromide (TetraPhimBr) was studied [30].

### 3.6. Differential Scanning Calorimetry (DSC)

**Figure 8:**
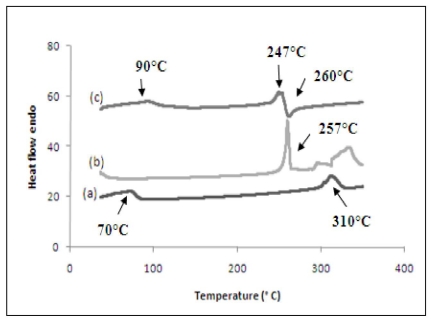
Differential scanning calorimetry curves of (**a**) β-CD; (**b**) PhenmimBr; and (**c**) β-CD-PhenmimBr.

DSC can be used for the recognition of inclusion complex. When guest molecules are embedded into β-CD cavity their melting, boiling or sublimating points generally shifts to different temperatures or disappears. The DSC results are presented in Figure 8 (a) β-CD, (b) PhenmimBr and (c) β-CD-PhenmimBr. DSC curve of β-CD-PhenmimBr is different from its precursor molecules, β-CD and PhenmimBr which further proves the formation of the inclusion complex [38]. As seen from Figure 8a, β-CD shows two strong endothermic peaks at 70 °C and 310 °C, the former due to elimination of the high-energy water molecules from β-CD cavity, the latter due to the decomposition of β-CD molecule. DSC curve of PhenmimBr exhibited a sharp endothermic peak at 257 °C which corresponds to the melting point of PhenmimBr as shown in Figure 8b. In the DSC curve of β-CD-PhenmimBr, the endothermic peaks characteristic of PhenmimBr and β-CD are slightly shifted. Referring to Figure 8c, the peak which is nearly identical to the melting point of pure PhenmimBr has shifted to lower temperature at approximately 247 °C. The other peak corresponding to β-CD shifts to a higher temperature around 90 °C which could be explained by the fact when PhenmimBr enters the cavity, the energy of the water molecules in the cavity changes. But surprisingly, a new exothermic peak at 260 °C, which did not exist in any other DSC curve, appears. The presence of this new peak is evidence for formation of the inclusion complex [32].

### 3.7. Scanning Electron Microscopy (SEM)

**Figure 9:**
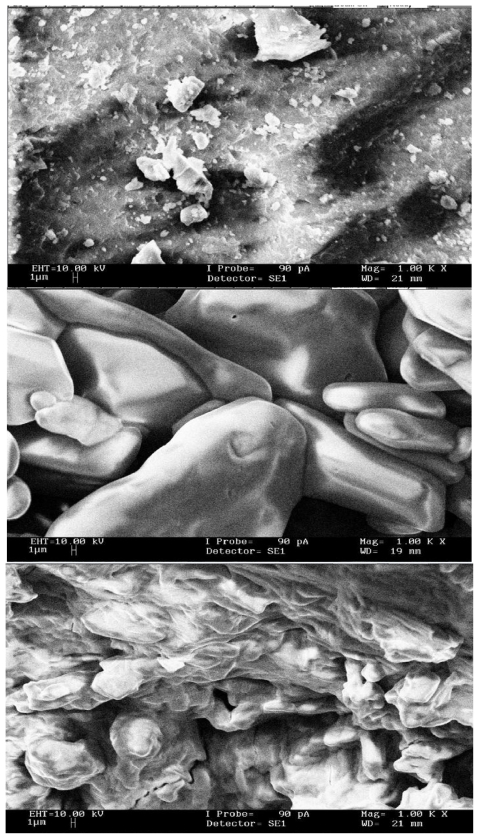
Scanning Electron Microscope photographs of (**a**) β-CD; (**b**) PhenmimBr; and (**c**) β-CD-PhenmimBr.

All the microscopic morphological structures were performed using Scanning Electron Microscope (SEM). SEM of β-CD revealed a “shrinked” crystal structure. It exhibited loss of sphericity, smooth surface and reduced size of particles as shown in Figure 9a. The PhenmimBr appeared as irregularly shaped crystals as shown in Figure 9b. As seen from Figure 9c, β-CD-PhenmimBr exhibited a totally different crystalline structure, which was not comparable with the morphology of pure β-CD and PhenmimBr, thus confirming the formation of β-CD-PhenmimBr. A drastic change in particle shape and morphology, although not conclusive but it supports the apparent interaction between β-CD and PhenmimBr in solid-state [39].

### 3.8. UV-Visible Spectroscopy

**Figure 10:**
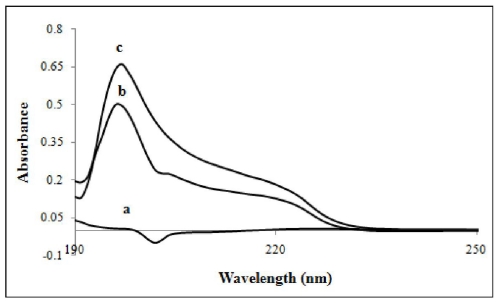
Absorption spectra of (**a**) β-CD; (**b**) PhenmimBr; and (**c**) β-CD-PhenmimBr with [PhenmimBr]: 0.01 mM and [β-CD]: 0.0032 M; at pH 7, *T* = 25 °C.

**Figure 11:**
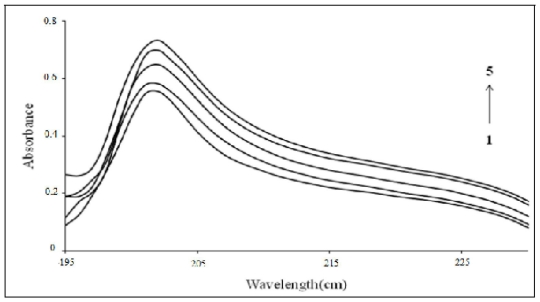
Absorption spectra of PhenmimBr with various concentration of β-CD at pH 7, *T* = 25 °C. From lines 1 to 5: 0 M; 0.001 M; 0.002 M; 0.003 M and 0.005 M.

The absorption spectra of β-CD-PhenmimBr complex, PhenmimBr and β-CD were recorded with accordance to procedure 2.4.1. The obtained result shows that β-CD had no absorption in the range of 190–250 nm. PhenmimBr was quite similar to β-CD-PhenmimBr in absorption spectrum shape, but the absorbance of β-CD-PhenmimBr was higher than that of PhenmimBr at 200 nm as shown in Figure 10. The influence of the β-CD concentration on PhenmimBr was also studied. As can be seen from Figure 11, addition of β-CD had caused a noticeable increase in the absorption intensity. This is because upon inclusion in the β-CD cavity, generally the absorbance of the guest molecule will be enhanced due to shielding of the excited species from non-radiative processes occurring in the bulk solution and also due to increase in molar absorption coefficient of the inclusion complex (β-CD-PhenmimBr).

#### 3.8.1. Stoichiometry of the Complex and Formation Constant

**Figure 12:**
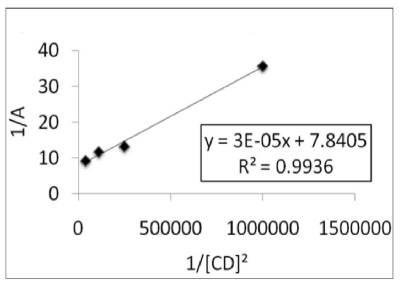
Reciprocal plot of 1/Absorbance *versus* 1/[β-CD]^2^.

The formation constant is the most important parameter in the study of inclusion behavior of β-CD. The formation constant for the inclusion complex has been determined by analyzing the changes in the intensities of absorption with different β-CD concentrations. The formation constant is denoted by K and the stoichiometric ratio of the β-CDPhenmimBr was determined using the modified Benesi-Hildebrand equation [40]. The K value was calculated by dividing the slope with the intercept of straight line obtained from the double reciprocal plot. A good linear relationship was obtained when 1/Absorbance is plotted against 1/β-CD^2^ with *R^2^* = 0.9936 for β-CD-PhenmimBr (Figure 12), indicating that the stoichiometric ratio of the complex formed is 2:1. The apparent formation constant is determined to be 2.61 × 10^5^ mol^−2^ L^2^. The dicationic IL (PhenmimBr) have higher formation constant value compared to monocationic ILs with reference to the tabulated results obtained by He *et al*. [28]. The formation constant for BmimCl and OmimBr is 8.16 and 672 L/mol respectively [28] which is lower compared to the value obtained for PhenmimBr. It is quite conclusive that dicationic IL have a greater tendency to bind strongly with β-CD than monocationic ILs. Furthermore, we suggest that the dicationic IL tends to form inclusion complex in 2:1 ratio due to the presence of two imidazolium rings meanwhile monocationic ILs have greater tendency to form 1:1 inclusion complex.

#### 3.8.2. Inclusion Structure

**Figure 13:**
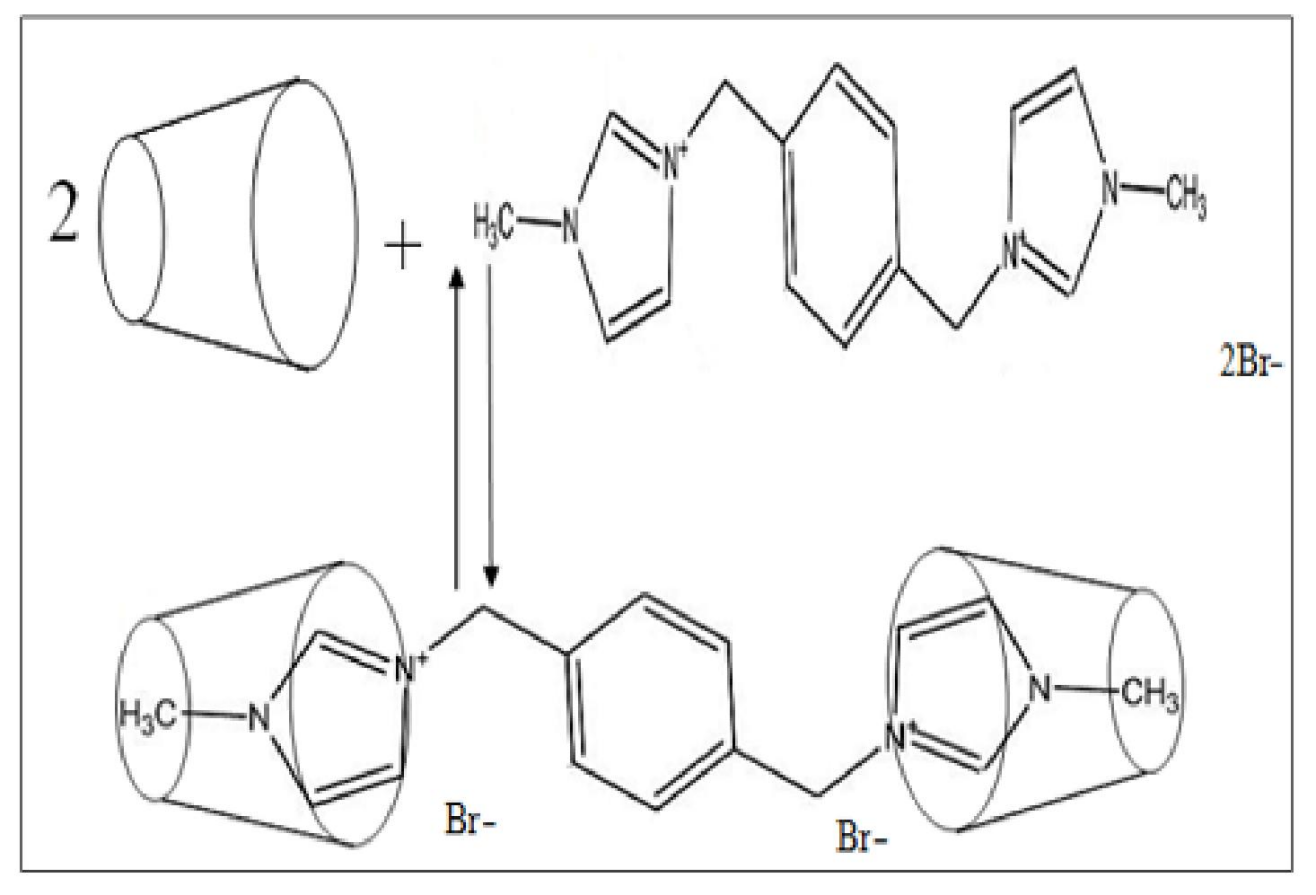
Proposed structure for possible inclusion mechanism.

The possible inclusion mechanism is proposed from the stoichiometric ratio (2:1) obtained. Furthermore, based on the ^1^H NMR and COSY results, the proposed structure of β-CD-PhenmimBr is presented in Figure 13. Based on our findings, it is very obvious that the imidazolium ring has entered the cavity of β-CD. But in contrast, He *et al.* recently had claimed that only the alkyl side chain of the IL interacts with β-CD while the imidazolium ring does not interact [28]. This discrepancy can be explained by the fact that the hydrophobic interaction and relatively larger size of the dicationic IL, PhenmimBr plays an important role in the inclusion process. The cation without long hydrocarbon chain can be included into the cavity of β-CD. However the key problem is the location of anion. It has been discovered that perhaps, anion dissociates near β-CD molecules.

## 4. Conclusion

The inclusion behavior of β-CD with dicationic IL, PhenmimBr has been investigated. The results obtained by different characterization techniques clearly indicates that the kneading method leads to the formation of a new product (β-CD-PhenmimBr), with properties different from those of originating host (β-CD) and guest molecules (PhenmimBr). Based on the FT-IR, ^1^H NMR and COSY spectra, it is found that the imidazolium ring of PhenmimBr has entered the hydrophobic cavity of β-CD. The dicationic IL (PhenmimBr) exhibits higher formation constant value (2.61 × 10^5^ mol^−2^ L^2^) compared to monocationic ILs. This suggests that the imidazolium rings in dicationic IL binds strongly with the hydrophobic cavity of β-CD which enables it to be applied in separation studies. Currently we are investigating the possibility of this complex as adsorbent for solid phase extraction.

## Figures and Tables

**Table 1:** Chemical shifts (δ) of β-CD, PhenmimBr and β-CD-PhenmimBr.

	β-CD	PhenmimBr	β-CD-PhenmimBr	

	δ	δ	δ	Δδ
H1	4.8191		4.8094	−0.0097
H2	3.3144		3.2955	−0.0189
H3	3.6402		3.5987	−**0.0415**
H4	3.3607		3.3504	−0.0103
H5	3.6006		3.5591	−**0.0415**
H6	3.6469		3.6189	−0.0280

Ha		3.8532	3.8410	−0.0122
Hb		9.3449	9.2430	−**0.1019**
Hc		5.4598	5.4226	−0.0372
Hd		7.8243	7.7633	−**0.0610**
He		7.7291	7.6974	−0.0317
Hf		7.4948	7.4637	−0.0311
